# SAG-UPS attenuates proapoptotic SARM and Noxa to confer survival advantage to early hepatocellular carcinoma

**DOI:** 10.1038/cddiscovery.2015.32

**Published:** 2015-09-28

**Authors:** S C Chang, W Q W Choo, H C Toh, J L Ding

**Affiliations:** 1 Department of Biological Sciences, National University of Singapore, 117543, Singapore; 2 NUS Graduate School for Integrative Science and Engineering, National University of Singapore, 117543, Singapore; 3 Division of Medical Oncology, National Cancer Centre Singapore, 169610, Singapore

## Abstract

Hepatocellular carcinoma (HCC) is a deadly cancer because of its commonly late diagnosis and limited treatment options. SAG (sensitive to apoptosis gene)-dependent UPS (ubiquitin–proteasome system) is a key switch between immune-mediated apoptosis and overactivation-mediated protumorigenesis, prompting us to hypothesize that SAG-UPS modulates chronic inflammation-induced tumorigenesis. Here, we investigated the molecular mechanism by which SAG-UPS regulates death/survival of liver cancer cells. By retrospective studies, we found reciprocal expressions of anti-/proapoptotic factors: SAG/SARM and SAG/Noxa in human primary HCC tissues – the antiapoptotic SAG was significantly upregulated whereas the proapoptotic SARM and Noxa were markedly downregulated, suggesting their involvement in hepatocarcinogenesis. Upregulated SAG-UPS effectively manipulates the levels of high-molecular-weight ubiquitinated SARM and Noxa in carcinoma tissues compared with corresponding normal tissues. *SAG*-overexpressing HCC cell lines display reduced SARM and Noxa (but not Bcl-2, Bax and Bcl-xL), suggesting that SARM and Noxa are specific substrates of SAG-dependent ubiquitination. *SARM* overexpression activated caspase-3 and caspase-9, reducing cell viability. *SAG* knockdown significantly elevated apoptosis with increased cytosolic cytochrome *c*, confirming SAG-mediated antiapoptosis in HCC. *SAG* overexpression stimulated protumorigenic cytokines, IL-1*β*, IL-6 and TNF, but not antitumorigenic IL-12p40 and anti-inflammatory IL-10. This is consistent with higher proinflammatory cytokines (IL-1*β*, IL-6 and TNF) in hepatoma compared with healthy tissues. Altogether, early stage-upregulated SAG-UPS exacerbates hepatocarcinogenesis progression, through: (1) ubiquitination-mediated degradation of proapoptotic SARM and Noxa; and (2) production of protumorigenic cytokines that induce a protumorigenic microenvironment, conferring survival advantage to HCC cells. Thus, we propose SAG-UPS to be an early diagnostic marker for HCC, and a potential target for therapeutics development.

## Introduction

Hepatocellular carcinoma (HCC) accounts for >80% of primary and malignant liver tumors,^1^ being the third most common cause of cancer deaths and the fifth most frequent malignancy worldwide.^2,3^ HCC has a poor prognosis, as reflected in its low 5-year survival rate of only ~10%.^4^ Unlike other cancers, HCC is especially refractory to therapy because it is often detected late and the efficacy of existing therapeutics is limited. Current diagnostic tools of HCC are based on radiologic imaging, serum *α*-fetoprotein (AFP) levels and histology.^5^ However, these tools have variable effectiveness for early diagnosis of HCC. The major limitation of cross-sectional imaging is its inability to detect tumors below 1 cm in diameter. Serological tests suffer poor sensitivity and specificity at the early stage of HCC, as the AFP level is associated with both HCC and chronic liver disease.^6,7^ There is therefore a great urgency to improve early diagnosis of HCC, and also to identify and validate new potential therapeutic targets.

The major risk factors for the development of HCC are hepatitis B/C viruses (HBV/HCV), nonalcoholic steatohepatitis and alcohol,^8,9^ presumably because of their effects on DNA damages via inflammation activation and reactive oxygen species (ROS) stimulation.^8^ Long-term and persistent infection with HBV/HCV are major contributory factors toward the development of HCC.^10^ However, the fundamental mechanisms that link chronic inflammation-induced immune responses to cancer biology are unclear. SAG (sensitive to apoptosis gene) is recently reported to be a key regulator of immune overactivation and protumorigenesis.^11^ Under an infection condition, SAG-dependent ubiquitin–proteasome system (UPS) ubiquitinates the proapoptotic Bax and SARM (sterile *α* and HEAT/armadillo-motif-containing protein) but not antiapoptotic Bcl-2, hence providing survival advantage to macrophages to fight the infection. On the other hand, Noxa, a proapoptotic factor that is characterized in several human cancer cell lines,^12^ is regulated by SAG-UPS.^13^ *SAG* overexpression has been observed in liver cancer, although in one sample.^13^ However, no information is thus far available on the potential involvement of key apoptotic factors regulated by SAG-UPS, particularly from HCC patient samples.

We previously reported that SAG-UPS manipulates the life and death of macrophages by modulating the balance between certain anti- and proapoptotic proteins, and regulating the pro- and antitumorigenic cytokines, thus creating a protumorigenic microenvironment.^11^ In the present work, we explored the contribution of SAG-UPS to liver cancer survival. We found that the levels of proinflammatory cytokines (IL-1*β*, IL-6, TNF and IL-12p40) and anti-inflammatory cytokines (IL-10) in the primary HCCs correlate with chronic liver inflammation. Based on our earlier findings, we examined the expression profiles of specific apoptotic proteins in human primary HCC tissues. We showed SAG to be highly expressed in the initial stage of hepatocarcinogenesis. Furthermore, Noxa and SARM, which are functional proapoptotic factors in liver cancer, are regulated by SAG-UPS. *SARM* overexpression (or *SAG* knockdown) markedly reduced the survival advantage of HCC cell lines. The protumorigenic potential of SAG was reflected in pro- and antitumorigenic cytokine levels, suggesting that SAG could be targeted to downregulate HCC tumorigenesis. Our findings provide novel insights into how SAG-UPS confers antiapoptosis strategy, and deregulates cell death /survival, in a protumorigenic microenvironment that promotes liver cancer progression.

## Results

### Clinical status of HCC patients

Our previous study has suggested that SAG-UPS plays a role in the life/death of macrophages during infection-mediated inflammation and it induces a protumorigenic microenvironment. To explore the clinical significance of SAG-UPS in response to infection and disease progression, here we performed a retrospective study on the impact of SAG on human primary HCC tissues. Table 1 summarizes the clinicopathological parameters of the HCC patient liver tissue samples. Clinical information was obtained from medical records of SingHealth Tissue Repository, Singapore. The age of the HCC patients ranged from 30 to 70 years, with a mean of 60 years. The HBV infection status was based on the hepatitis B surface antigen (HBsAg). HBV infection is the most common underlying condition among HCC patients. Out of 11 patients, 9 were HBsAg positive. The clinical stages were based on clinical tumor–node–metastasis (TNM): stage II (*n*=5), stage IIIA (*n*=2) and stage IIIB/C (*n*=4).

### Primary HCC tissues express reciprocal profiles of SAG/SARM and SAG/Noxa

**Figure 1:**
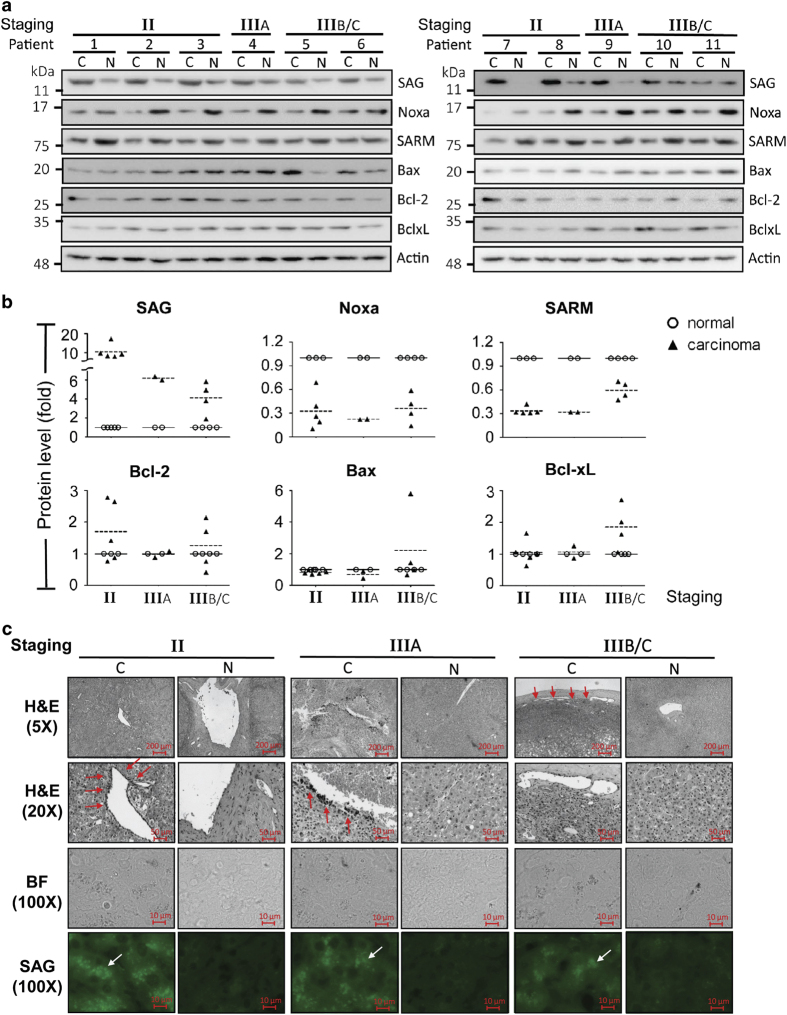
Primary HCC tissues show reciprocal expression profiles of SAG/SARM and SAG/Noxa. The expression profiles of various established and recently identified apoptotic factors were characterized in primary HCC tissues. Total protein extracts of HCC tissues and adjacent normal tissues from 11 patients were examined by (**a**) immunoblotting that were quantified (**b**). In general, higher levels of SAG were observed in carcinoma tissues compared with corresponding normal tissues (*P*<0.01), suggesting its involvement in liver cancer. In contrast, proapoptotic Noxa was found to be significantly decreased in carcinoma tissues (*P*<0.01). Similarly, lower SARM was observed in primary HCC tissues (*P*<0.01), suggesting its role as a proapoptotic factor in liver cancer. Expression of SAG *versus* SARM and SAG *versus* Noxa appears to be reciprocal between normal and carcinoma tissues in each individual, suggesting that Noxa and SARM may be under the regulation of SAG-dependent UPS-mediated protein degradation in liver cancer. Expression levels of apoptotic factors were normalized against *β*-actin expression. C, carcinoma tissues; N, normal tissues. **P<0.01. (**c**) H&E and immunofluorescence staining of liver sections from HCC tissues and normal counterparts. Inflammatory cell infiltration is indicated with red arrows. In tumor tissues, the highly expressed levels of SAG appear as punctate clusters (white arrow), compared with corresponding normal tissues.

We characterized the expression profiles of a panel of apoptotic factors in primary HCC, with a view to their potential involvement in hepatocarcinogenesis. In total, six apoptotic factors were examined, including SAG, Noxa, SARM, Bax, Bcl-2 and Bcl-xL, based on our earlier observations.^11^ Furthermore, consistent with recent findings in lung cancer,^13^ the antiapoptotic SAG protein in the HCC tissues was remarkably higher than that in the adjacent noncancerous hepatic tissues (*P*<0.01). In contrast, the levels of proapoptotic Noxa and SARM were significantly lower in HCC than in the healthy controls (*P*<0.01) (Figures 1a and b). The protein levels of Bax, Bcl-2 and Bcl-xL did not show a clear or consistent trend between HCC and the corresponding normal tissues among all tested samples. We observed that Bcl-2 was either upregulated in some HCC cases (patients 5/6/7/8) or downregulated in others (patients 10/11). Similarly, Bax was found to be mainly upregulated in certain HCC samples (patients 5/6) and showed no significant differences in other HCC samples. Overall, our results indicate reciprocal expression profiles between SAG/Noxa and SAG/SARM in primary HCC, suggesting that SAG might regulate liver cancer progression by opposing the roles of Noxa and SARM. To show the interpatient changes in the expression profiles of apoptotic factors in HCC samples, we also reorganized the western blot image according to tumor stages (Supplementary Figure 1). To confirm the expression status of SAG in human liver tissues, we performed immunofluorescence staining of SAG. Consistent with immunoblotting analysis, Figure 1c shows more strongly expressed SAG in punctate appearance in HCC tissues, whereas the adjacent normal tissues expressed SAG minimally. Furthermore, H&E staining shows increased infiltration of inflammatory cells in HCC tissues (red arrows), indicating higher inflammation levels in carcinoma tissues.

First, Noxa and SARM are reported to be proapoptotic,^11,13^ and, second, SAG (antiapoptotic) displays reciprocal levels of expression in primary macrophages during infection.^11^ These findings prompted us to hypothesize that in liver cancer, SAG probably downregulates SARM and Noxa, thus promoting cancer cell survival. In clinical TNM staging (Table 1), we found that SAG expression in the early stage (II) was 10-fold higher than normal controls (*P*<0.01), whereas in the advanced stages (IIIA and IIIB/C) the SAG levels were reduced to 6- and 4-fold, respectively, relative to normal controls (*P*<0.01). The high level of SAG at the earlier stage of HCC suggests its potential as an early diagnostic marker. Interestingly, we found that SARM expression is increased at the later stages of liver cancer, suggesting that the reciprocal relationship of SAG/SARM expression may be associated with the progression of hepatocarcinogenesis. Furthermore, based on our observations here and in previous findings,^11^ it is conceivable, from the early increase in SAG expression, that SAG initiates and promotes liver cancer, probably by downregulating Noxa and SARM via protein degradation.

### SAG ubiquitinates SARM and Noxa in primary HCC

**Figure 2:**
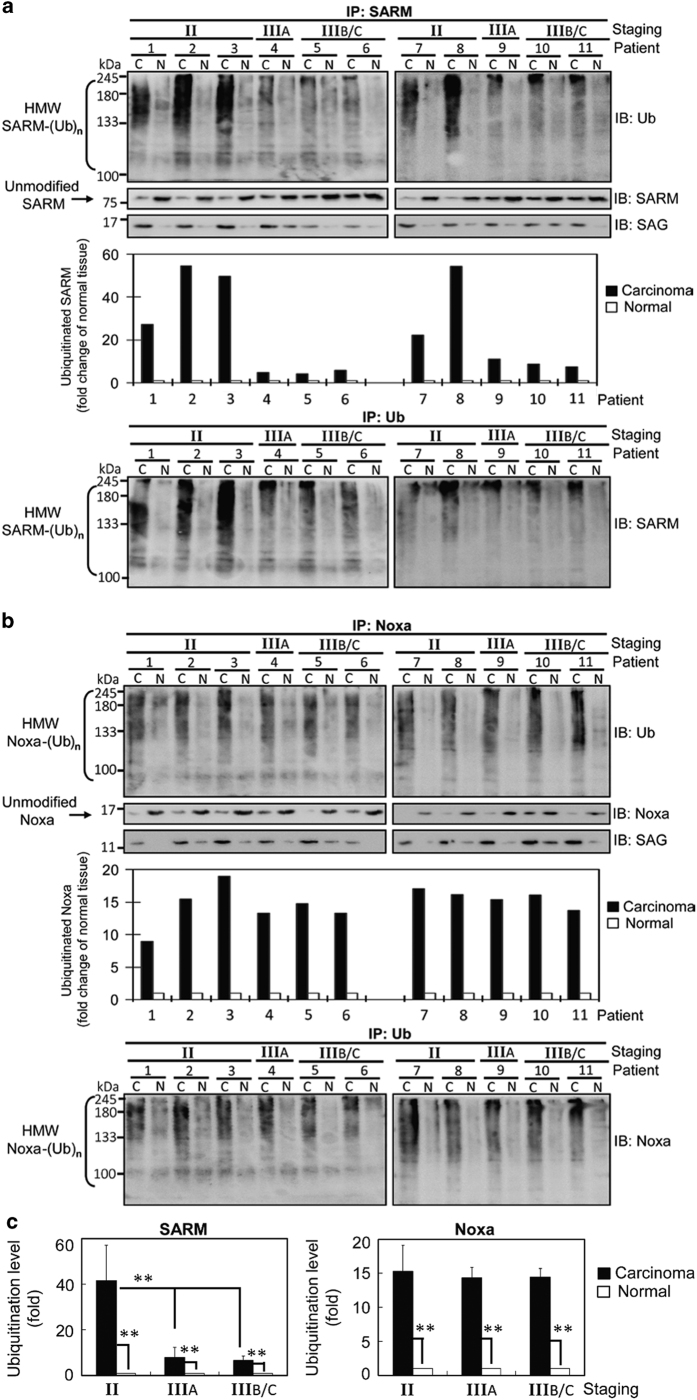
SAG regulates proapoptotic SARM and proapoptotic Noxa by ubiquitination of these proteins in the primary HCC tissues. To further confirm whether SARM and Noxa are under the regulation of SAG-dependent UPS, total protein extracts from primary tissues were co-immunoprecipitated (co-IP) with (**a**) SARM or (**b**) Noxa antibody, followed by immunodetection using ubiquitin, SARM, Noxa or SAG antibody. Co-IP shows that both SARM and Noxa pulled down SAG, confirming the interaction between SAG and SARM (or Noxa) in primary HCC. A densitometric analysis of ubiquitinated SARM or Noxa normalized with corresponding normal control tissues is plotted. (**c**) Quantitation of the ubiquitination levels of SARM and Noxa. We found a significant increase in ubiquitination of SARM and Noxa in carcinoma tissues, compared with corresponding normal tissues (*P*<0.01). Interestingly, consistent with highly expressed SAG, we found a significant increase in Ub-SARM at early stage (II) of carcinoma (*P*<0.01), suggesting the potential of SAG as an early diagnostic marker. HMW SARM-(Ub)n, high-molecular-weight ubiquitinated SARM; HMW Noxa-(Ub)n, high-molecular-weight ubiquitinated Noxa. Arrows indicate unmodified (un-ubiquitinated) Bax or SARM. Data are representative of means±S.D. (*n*=3).

To elucidate the mechanism of action of SAG-UPS that leads to the progress in liver cancer, the ubiquitination status of SARM and Noxa was retrospectively examined in tissues from different stages of liver cancer. Total protein extracts from primary tissues were immunoprecipitated with SARM or Noxa antibody, followed by ubiquitin-specific immunodetection. To confirm that the specificity of ubiquitination was due to SARM (or Noxa), a converse immunoprecipitation (IP) with anti-ubiquitin antibody followed by immunoblotting with SARM (or Noxa) was also performed. Figure 1a shows the corresponding immunoblots (before IP) with whole tissue lysates that were sequentially probed with antibodies against SAG, BAX, SARM, Noxa, Bcl-2 and Bcl-xL. The results show the presence of these proteins in the whole tissue lysates. A negative control immunoprecipitation was performed with an isotype rabbit IgG. Co-IP showed a significant increase in both the high-molecular-weight ubiquitinated SARM and Noxa (HMW SARM-(Ub)n and HMW Noxa-(Ub)n) in carcinoma tissues, compared with corresponding normal control tissues (Figures 2a and b). Among the different stages of carcinoma tissues examined, a 15-fold increase in Ub-Noxa was observed in HCC tissues (Figure 2c, *P*<0.01), whereas Ub-SARM was increased over a range of 7–40-fold (Figure 2c, *P*<0.01). Both of the co-IP of SARM and Noxa showed pulldown of SAG in the immunocomplexes, indicating that SAG physically interacts with SARM and Noxa. Elevated SAG was associated with increased ubiquitination of SARM (or Noxa) that corresponded to decreased unmodified proteins in carcinoma tissues (see arrows). Figure 2c depicts a 40-fold rise in Ub-SARM at the early stage (II) of HCC, compared with a 7-fold rise in the advanced stages (IIIA and IIIB/C) (*P*<0.01), consistent with the early increase of 10-fold of SAG in stage II (Figure 1). This suggests that the regulation of SAG/SARM is probably exerted by SAG-UPS as the disease progresses. Taken together, our findings affirm that SAG-UPS interacts with and ubiquitinates SARM and Noxa in hepatocarcinogenesis.

### *SAG* overexpression attenuates proapoptotic Noxa and SARM in HCC cells

**Figure 3:**
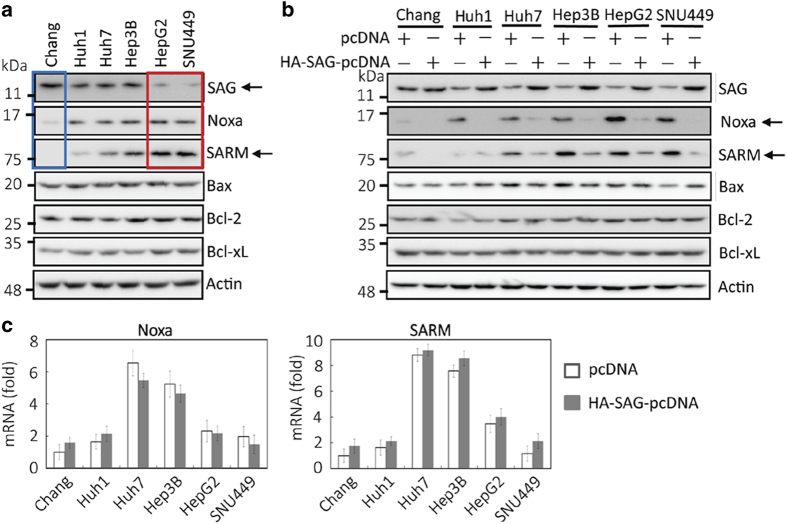
*SAG* overexpression in HCC cells attenuates proapoptotic Noxa and SARM. To further confirm the critical regulator(s) in liver cancer, we characterized the expression profiles of proapoptotic (Noxa, SARM and Bax) and antiapoptotic (SAG, Bcl-2 and Bcl-xL) factors in six human HCC cell lines. Consistent with primary HCC, immunoblottings showed reciprocal relationship in both Noxa/SAG and SARM/SAG in HCC cell lines (**a**, blue and red box). To confirm whether Noxa and SARM are regulated by SAG-dependent protein degradation, HA-SAG was transiently transfected and overexpressed in HCC cells, followed by immunoblotting analysis (**b**) and real-time PCR (**c**). *SAG* overexpression attenuated both Noxa and SARM proteins throughout six tested HCC cell lines (shown as arrows), but not Bcl-2 and Bcl-xL. Bax was found to be decreased in Huh7 and Hep3B cells, and increased in SNU449, when SAG was overexpressed. Real-time PCR affirms that attenuated Noxa and SARM under SAG overexpression is due to a post-transcriptional event, suggesting the involvement of SAG-dependent UPS. Actin was used as a loading control for immunoblotting. qRT-PCR values are normalized using human *β*2-microglobulin gene and presented as means±S.D. (*n*=3).

To elucidate how SAG-UPS regulates Noxa and SARM in liver cancer, we characterized six different human HCC cell lines for their expression profiles of apoptotic factors under various stimulatory challenges. Based on our earlier findings^11^ and the observations in this study, we assessed the endogenous levels of SAG, Noxa, SARM, Bax, Bcl-2 and Bcl-xL by immunoblotting (Figure 3a). Consistent with primary HCC tissues, we found reciprocal relationship in SAG/SARM and SAG/Noxa in the cell lines. Chang cells showed high level of SAG but low levels of Noxa and SARM (blue box). On the contrary, HepG2 and SNU449 cells showed low SAG and high SARM (red box). Huh1, Huh7 and Hep3B displayed intermediate levels of SAG, SARM and Noxa. These data highlight the intratumoral heterogeneity in liver cancers, supporting the fastidious nature of the disease and hence the difficulty in finding a perfect treatment for hepatoma.

The metastatic potential of the six HCC cell lines was further examined by measuring CD44 and Twist1, the two mesenchymal markers.^14–16^ However, there was no apparent correlation between the reciprocal apoptotic profiles of SAG/Noxa and SAG/SARM and the metastatic potential in these HCC cell lines (Supplementary Figure 2). Nevertheless, as SAG is a key component of E3 ubiquitin ligase that is responsible for ubiquitin-associated protein degradation, we next determined whether Noxa and SARM are the direct substrates of SAG-dependent protein degradation. HA-SAG was transiently transfected and overexpressed in the HCC cells, followed by immunodetection of the apoptosis proteins (Figure 3b). The transfection efficacy was confirmed by SAG immunodetection. Unlike Noxa and SARM (Figure 3b, arrows) that are consistently reduced in all six HCC cell lines, *SAG* overexpression did not cause a clear change in the levels of Bcl-2 and Bcl-xL. In this regard, there was no clear trend – downregulation of Bax was seen with *SAG* overexpression in Huh7 and Hep3B cells, whereas upregulation of Bax was observed in SNU449 cells. Immunoblotting with anti-HA antibody further demonstrates the specificity of the assay (Supplementary Figure 3). Real-time PCR affirmed that attenuation of Noxa and SARM was not because of changes in their mRNA levels, rather it was attributable to a post-transcriptional event, suggesting the involvement of SAG-UPS degradation of SARM and Noxa proteins (Figure 3c). Together with the data from primary HCC, it is conceivable that both Noxa and SARM (but not Bax, Bcl-2 and Bcl-xL) are specific substrates of SAG-dependent ubiquitination. These observations also corroborate that Noxa and SARM would have normally elicited proapoptotic activities against liver cancer cells if not for the dominant antiapoptotic role of SAG that ubiquitinates and degrades Noxa and SARM proteins.

### *SARM* overexpression induces intrinsic apoptosis in HCC cell lines

**Figure 4:**
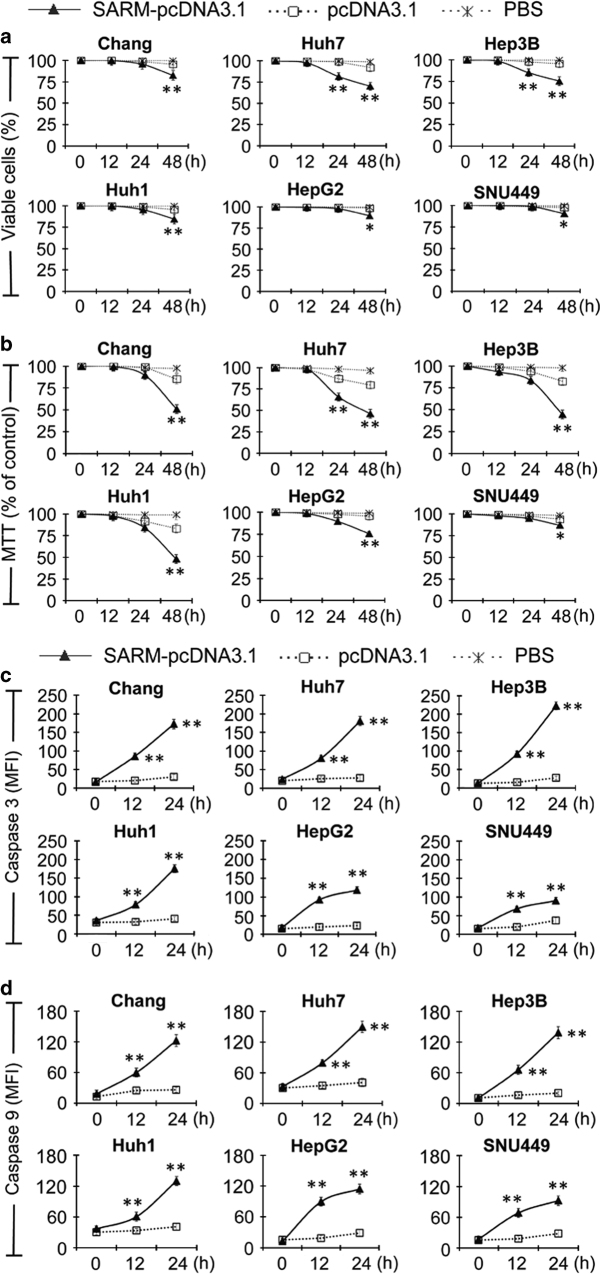
SARM acts as an intrinsic proapoptotic factor in HCC. To elucidate the functional roles of SARM in liver cancers, we tested the cell viability and caspase activity in *SARM*-overexpressed HCC cell lines. Transfected cells were examined by (**a**) Trypan blue exclusion assay, (**b**) MTT assay, (**c**) caspase-3 cleavage and (**d**) caspase-9 cleavage tests. We found that *SARM* overexpression reduced cell viability in all six HCC cell lines. In addition, *SARM* overexpression elevated the activities of caspase-3 and caspase-9, suggesting that SARM plays a proapoptotic role via intrinsic pathway in liver cancer. FACS histograms of caspase-9 and caspase-3 are shown in Supplementary Figure 5. Data are representative of means±S.D. (*n*=3). MFI, mean fluorescence intensity. **P<0.01.

The proapoptotic potential of Noxa is recently identified in HepG2 and Bel-7402 cells,^17^ whereas the role of SARM, although recently found to be proapoptotic in T cells^18^ and macrophages,^11^ remains unclear in malignant transformation. We speculated that an active suppression of the proapoptotic SARM might lead to tumor progression. This prompted us to further investigate the contribution of SARM in HCC cell death/survival. Thus, we monitored the cellular changes in HCC cell lines following *SARM* overexpression. The transfection efficacy was optimized with SARM-pcDNA3.1 and control pcDNA3.1 vector in the HCC cell lines (Supplementary Figure 4). Cell viability was examined by Trypan blue exclusion (Figure 4a) and MTT assay (Figure 4b). Over the time course of *SARM* overexpression, a significant drop in cell viability was observed, particularly with MTT assay. The results here suggest that SARM induces apoptosis in liver cancer cells. At 48 h post transfection, we observed >50% decline in mitochondrial activity in Chang, Huh7, Hep3B and Huh1 cells (*P<*0.01), whereas HepG2 showed an intermediate decrease of 25% (*P<*0.01). Compared with control cells transfected with pCDNA3.1 vector alone, *SARM* overexpression in SNU449 only resulted in minimal reduction in cell viability of 10% in Trypan blue exclusion (*P<*0.05) and 15% in MTT study (*P<*0.05). Among all HCC cells tested, SNU449 seems to be most resistant to cell death induced by *SARM* overexpression (Supplementary Figure 4). This appears to correlate negatively with the low SAG-UPS activity, indicating that there may be other parallel factors or mechanisms that facilitate SNU449 cell survival. The mechanism underlying the resistance potential of SNU449 cells to SARM-mediated killing needs to be examined in future. To better understand the regulatory signaling involved in HCC cell death due to *SARM* overexpression, we examined the caspase activities in *SARM*-overexpressing HCC cell lines. We found that SARM induced a significant increase in caspase-3 activity/cleavage in all cells tested (*P<*0.01, Figure 4c), confirming apoptosis in *SARM*-overexpressing HCC cells.

Consistently, SNU449 showed only moderate levels of caspase-3 activity by 24 h post transfection, whereas other HCC cells displayed higher caspase-3 activity. From 12 h post transfection onwards, *SARM* overexpression significantly elevated the activities of caspase-9 in HCC cells (*P<*0.01, Figure 4d), suggesting that SARM acts via intrinsic apoptosis in liver cancer. The reciprocal patterns of SAG *versus* SARM (or SAG *versus* Noxa) observed in both the primary HCC tissues and the cell lines support our hypothesis that SAG opposes SARM and Noxa activities during hepatocarcinogenesis. Therefore, we next investigated the effects of SAG on cell death/survival in HCC cells.

### *SAG* knockdown promotes intrinsic apoptosis in HCC

**Figure 5:**
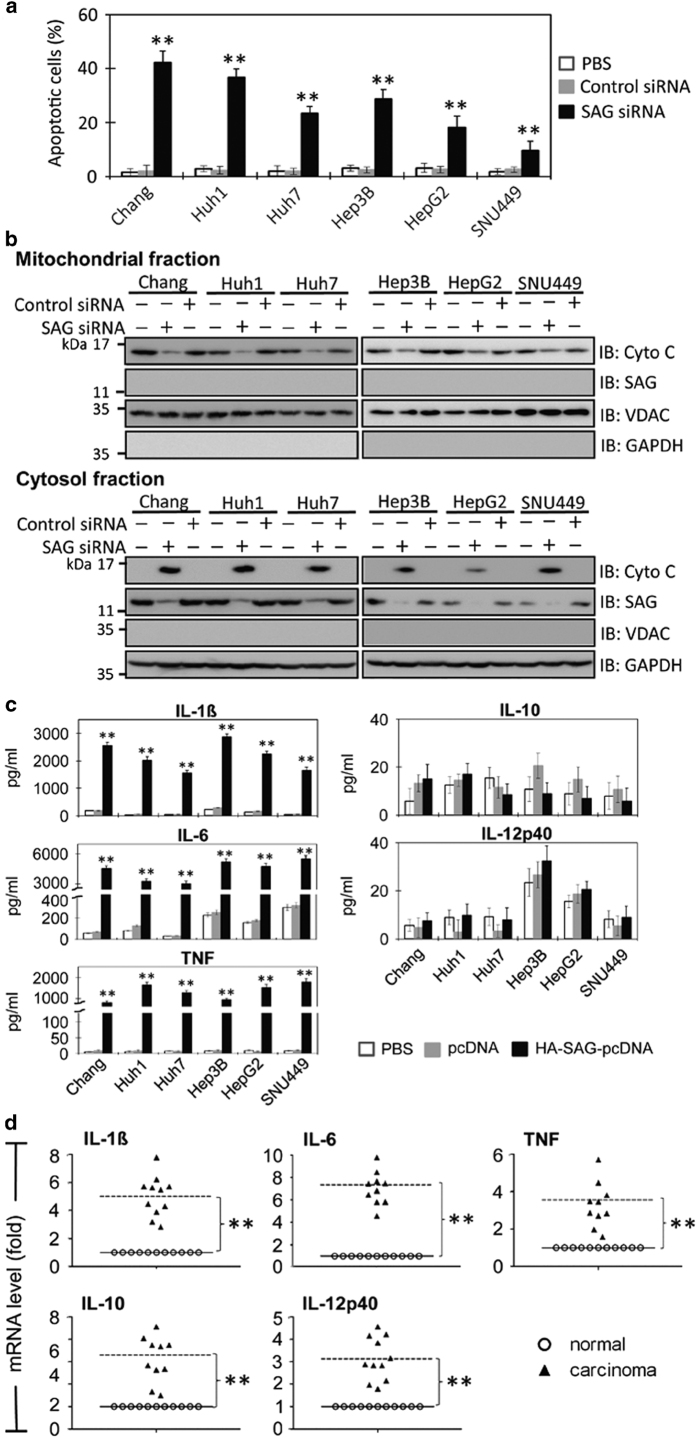
SAG acts as an antiapoptotic and protumorigenic factor in human HCC. Six human HCC cell lines were transfected with SAG siRNA (or control siRNA) for 24 h, followed by (**a**) apoptosis analysis and (**b**) cytochrome *c* release assay. Apoptotic cells were determined by double staining with Annexin V and 7AAD. FACS analysis showed a significant increase in early apoptotic cells (Annexin V^+^/7AAD^−^) in *SAG*-knockdown cells. Cytochrome *c* release indicates the level of intrinsic apoptosis that is increased in all *SAG*-knockdown HCC cells. (**c**) Six HCC cells were transfected with SAG-HA-pcDNA or Control pcDNA for 24 h. The levels of protumorigenic cytokines (IL-1*β*, TNF and IL-6), antitumorigenic cytokine (IL-12p40) and anti-inflammatory cytokine (IL-10) in the culture supernatants were then examined using ELISA (*n*=3). (**d**) The mRNA levels of the cytokines were measured by real-time PCR in primary HCC tissues. C, carcinoma tissues; N, normal tissues. **P<0.01.

FACS analysis (Supplementary Figure 6) showed that *SAG* knockdown significantly increased apoptosis in all six HCC cell lines (Figure 5a, *P*<0.01). At 24 h post siRNA treatment, early apoptosis was observed in 35-40% of Chang and Huh1 cells compared with 10–25% of Huh7, Hep3B, HepG2 and SNU449 cell lines. Control siRNA (or PBS)-treated cells showed minimal apoptosis, suggesting that the increased apoptosis of HCC cells is specifically induced by SAG silencing.

To affirm the relationship between SAG expression and the intrinsic apoptotic potency in liver cancer, we tested the release of cytochrome *c* in SAG-knockdown HCC cells. *SAG* sequence-specific siRNA knockdown was optimized in the six HCC cell lines, showing increased level of cytosolic cytochrome *c* at the expense of mitochondrial cytochrome *c* (Figure 5b). Thus, we propose that SAG is a functional antiapoptotic factor acting via the intrinsic pathway in HCC, and this might be mediated through an interplay in SAG *versus* SARM (or SAG *versus* Noxa).

### Protumorigenic SAG induces protumorigenic cytokines in HCC

Previously, we showed the potential of SAG-UPS in the recruitment of immune cells to a tumor microenvironment mimicked by infection–inflammation condition.^11^ In the current work, to better understand the effects by SAG in a neoplastic microenvironment, we tested the relationship between SAG and pro- and antitumorigenic cytokines in the HCC cell lines. Culture supernatants from the six cell lines were examined for five cytokines (IL-1*β*, IL-6, IL-10, IL12p40 and TNF, representing pro- and anti-inflammatory cytokines). We found that *SAG* overexpression significantly increased protumorigenic/proinflammatory cytokines, IL-1*β*, IL-6 and TNF, but not the anti-inflammatory IL-10 and antitumorigenic IL-12p40 (Figure 5c). The results support that SAG probably plays a protumorigenic effect by perturbing the fine balance between the pro- and antitumorigenic cytokines.

Next, we queried the status of the expression of these pro- and antitumorigenic cytokine genes in the liver tissues of the HCC patients. Coincidently, we found that protumorigenic/proinflammatory cytokines, IL-1*β*, IL-6 and TNF, along with SAG, were significantly upregulated in the primary carcinoma tissues (Figure 5d, *P*<0.01). Both the anti-inflammatory IL-10 and protumorigenic IL-12p40 that have been reported to be associated with chronic infection–inflammation^19,20^ were significantly raised in the primary HCC tissues (Figure 5d, *P*<0.01). This observation is clearly consistent with clinical presentation. In summary, our findings suggest the protumorigenic role of antiapoptotic SAG in HCC carcinogenesis, by (1) regulating the levels of proapoptotic SARM and Noxa and (2) imbalancing and immunomodulating the levels of pro- and antitumorigenic cytokines.

## Discussion

As a key component of SCF (Skp1–cullin–F-box proteins) E3 ubiquitin ligase, SAG is recently recognized as an anticancer agent in a pancreatic cancer system *in vivo.*^13^ However, the detailed molecular mechanism of action of SAG in cancer progression is hitherto unknown. Here, we explored the novel regulatory mechanisms of SAG-dependent UPS in HCC, a major form of deadly cancer worldwide. For the first time, we monitored the status of ubiquitination in human primary HCC tissues and delineated key components involved in different stages of hepatoma. We showed that SAG mediates degradation of SARM and Noxa but not Bax, Bcl-2 and Bcl-xL, thus identifying SARM and Noxa as specific proapoptotic proteins targeted by SAG-UPS, hence reducing their anticancer effect normally conferred by SARM/Noxa. Upregulated SAG in HCC tissues correlates with imbalanced anti-/proapoptotic proteins, in which proapoptotic proteins are ubiquitinated, hence providing survival advantage to cancer cells. There has been little improvement in the natural history of HCC because of late diagnosis in many cases and suboptimal systemic treatment of the disease in the advanced stage.^2,3^ Our findings here highlight SAG-UPS as a promising candidate for both early diagnosis and development of therapeutics.

The elevated expression of SAG was observed at the early stage of HCC (Figure 1), suggesting that SAG is related to the histological grade of HCC and is involved in the malignant transformation process. Supporting this hypothesis is gankyrin (known as 26S proteasome regulatory subunit p28 or p28^GANK^) that is also reported to have an early function in HCC pathogenesis. Gankyrin is known to contribute to the development of HCC by eliminating tumor suppressors p53, Rb and C/EBPa.^21,22^ In addition to gankyrin, there are other UPS components that are also highlighted in HCC, including Siah1 and Parkin.^23,24^ Thus, the complexity of UPS components, including SAG, may exert multiple and simultaneous roles during HCC development. Coincidentally, we found that both Ub-SARM and Ub-Noxa were outcomes of upregulated SAG-UPS. However, Ub-SARM (but not Ub-Noxa) appears to be associated with the HCC disease progression. This event also suggests the involvement of other components of SCF E3 ligases during hepatocarcinogenesis (for example, F-box that provides specificity to the tetrameric SCF E3 ligase).^25^ Hence, new avenues remain opened, for example, to identify specific F-box protein corresponding to the substrate of SAG-SCF E3 ligase.

**Figure 6:**
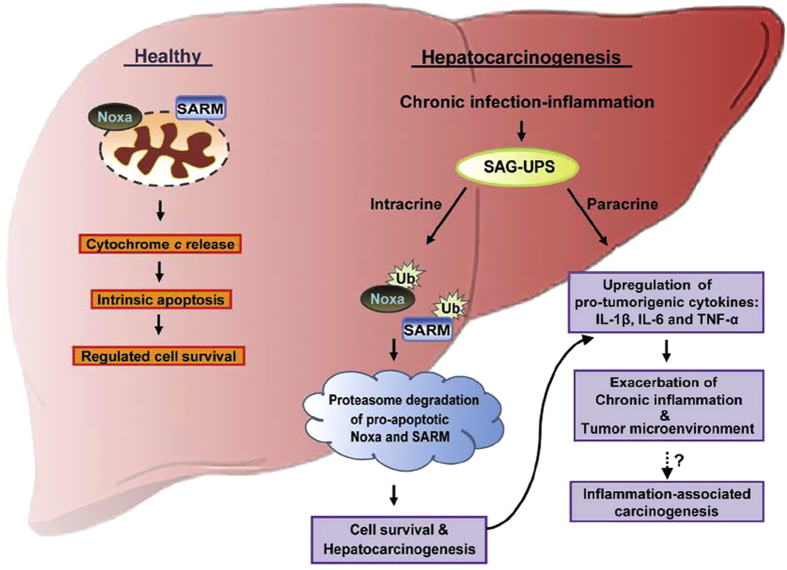
Schematic representation of SAG-dependent UPS linking chronic inflammation and liver cancer. Left and right sections represent healthy condition and hepatocarcinogenesis, respectively. Chronic infection by hepatitis viruses (HBV and HCV) is a major risk factor for the initiation and development of HCC. During infection, upregulated SAG-UPS via intracrine signaling attenuates proapoptotic Noxa and SARM by ubiquitination, leading to imbalanced anti-/proapoptotic factors. On the other hand, activation of SAG promotes protumorigenic IL-1*β*, TNF and IL-6 via paracrine signaling, thus exacerbating the tumor microenvironment. In summary, we hypothesize that SAG-dependent UPS is a key player in inflammation-associated carcinogenesis by manipulating the balance between pro-/antitumorigenic cytokines.

Chronic inflammation is now accepted as critical in fibrosis, cirrhosis and hepatocarcinogenesis.^10^ Although hepatic inflammation caused by long-term infection with hepatitis virus is implicated, the key components linking inflammation and liver cancer are only beginning to be unmasked. Chronic inflammation is characterized by continuously expressed cytokines and recruitment of immune cells to the liver. In consideration of our earlier study in macrophages,^11^ we postulate that overactivated SAG-UPS triggered by a protumorigenic microenvironment could be a key orchestrator of inflammation-associated carcinogenesis (Figure 6). SAG-UPS, in response to chronic infection via both intracrine and paracrine effects, may contribute to a vicious circle of chronic inflammation and carcinogenesis. Being a crucial member at the crossroad between inflammation and cancer, SAG appears to be an important modulator of proinflammatory/protumorigenic molecules (IL-1*β*, IL-6 and TNF). The tumor-associated macrophage-released cytokines favor a tumor microenvironment that initiates and stimulates the development of HCC, providing survival advantage to the cancer cells in HCC patients.^26^ In future, a better understanding of the molecular events underlying the relationship between cancer cells and their surrounding microenvironment would enable more realistic strategies for the development of therapeutic targets. In addition, although 9/11 of HCCs are HBV positive, we found that the two hepatoma cases with ‘unknown’ etiology also showed the involvement of SAG-UPS in progressive malignant transformation of liver cancer, hence suggesting the ubiquitous nature of SAG-UPS in cancer progression. This would need further confirmation in future through testing other forms of cancer.

In summary, our findings demonstrate for the first time the molecular mechanisms underlying how SAG-UPS regulates immune overactivation and protumorigenesis, linking immunology and cancer biology. SAG-UPS could be an efficient therapeutic target for developing immunomodulatory drug leads against autoimmunity, immunodeficiency diseases^11^ and cancer prevention (this study). Further work is warranted to explore potential contributions of SAG-UPS to the recruitment of immune cells to a tumor microenvironment in the background of chronic infection.

## Materials and Methods

### Tissue samples

Eleven cases of HCCs and their paired normal liver tissues (in total, *n*=22) were acquired from the National Cancer Center, SingHealth Tissue Repository (STR), Singapore, after clinical diagnosis of HCC was confirmed by biopsy and histological evaluation. Adjacent noncancerous liver tissues were obtained at least 2 cm away from the tumor node. All HCC patient samples were collected with informed consent (STR). For retrospective studies, liver tissues were immediately excised, frozen in liquid nitrogen and stored at −80 °C until processing. Each frozen tissue sample was sectioned and quickly homogenized with RIPA buffer (Thermo Scientific Pierce, Rockford, IL, USA; 1 ml per 20 mg of tissue). Protease inhibitors (complete EDTA-free cocktail, Roche, Mannheim, Germany) were added to RIPA buffer immediately before use. After a 30-min homogenization on ice, the samples were centrifuged at 10 000×*g* for 20 min at 4 °C. The supernatant was retrieved for further studies. The H&E sections were obtained from the SingHealth Pathology, Singapore. For immunofluorescence staining, paraffin-embedded tissues were sectioned at 5 *μ*m thickness, dehydrated and blocked in 3% donkey serum in PBS for 1 h at room temperature. Then, the sections were incubated with primary and secondary antibodies overnight and 1 h, respectively. The stained slides were observed under a microscope (Carl ZEISS Axio Observer Z1, Thornwood, NY, USA) and images were acquired using software (AxioVision Rel. 4.7, Thornwood, NY, USA).

### Human HCC cell lines and reagents

HepG2, Hep3B, Huh7 and SNU449 were grown in DMEM supplemented with 10% (vol/vol) fetal bovine serum. Chang and Huh1 cells were maintained in RPMI-1640 supplemented with 10% fetal bovine serum. To detect cytochrome *c* release, we prepared cytosolic and mitochondrial lysates of the cells. Briefly, the HCC cells were homogenized, and mitochondrial fractions were acquired by a mitochondria isolation kit (Thermo Fisher Scientific, Rockford, IL, USA). The primary antibodies used include: SAG polyclonal antibody (Abcam, Cambridge, MA, USA), Bcl-2 monoclonal antibody (Santa Cruz, Dallas, TX, USA), Bax polyclonal antibody (Cell Signaling, Danvers, MA, USA), Noxa polyclonal antibody (Santa Cruz), SARM monoclonal antibody (Cell Signaling), ubiquitin monoclonal antibody (Santa Cruz), GAPDH monoclonal antibody (Santa Cruz), VDAC polyclonal antibody (Cell Signaling), cytochrome *c* monoclonal antibody (BD Biosciences, San Jose, CA, USA), Alexa 488-conjugated secondary antibody (Invitrogen, Grand Island, NY, USA) and a loading control, *β*-actin (Sigma, St Louis, MO, USA).

### SARM-V5 overexpression, *SAG* knockdown and *HA-SAG* overexpression

SAG siRNA (silencer select pre-designed siRNA) and control (scrambled) siRNA were purchased from Ambion (Pittsburgh, PA, USA) and Invitrogen, respectively. SARM-V5-pcDNA3.1 and pcDNA3.1 plasmids were used for SARM overexpression assay. HA-SAG-pcDNA3 and pcDNA3 plasmids were used for SAG overexpression study. The transfection was conducted using X-tremeGENE Transfection Reagent (Roche) with 90 pmol of siRNAs or Lipofectamine 2000 Reagent (Invitrogen) with 2.5 *μ*g DNA plasmids per well of a 6-well plate. The efficacy of transfection was determined by immunodetection of SARM-V5 protein (Supplementary Figure 3) and SAG protein (Figures 3b and 5b).

### Co-IP and immunodetection

Co-IP study was performed with human primary tissues (*n*=22). Tissues were lysed as mentioned above (Tissue samples). The lysates were precleared by incubating with protein G Sepharose (GE Healthcare, Uppsala, Sweden) at 4 °C for 2 h. The supernatant was incubated overnight at 4 °C with Noxa, SARM or ubiquitin antibody, followed by incubation of protein G Sepharose for 3 h. The immunocomplexes were washed, resuspended in Laemmli buffer and boiled at 95 °C for 10 min. The co-IP products were immunoblotted and probed sequentially with antibodies to SAG, Noxa, SARM or ubiquitin.

### Detection of released cytokines

To determine the effects of *SAG* overexpression on cytokine production, HCC cells were transiently transfected with HA-SAG or control empty vector for 24 h. The concentrations of IL-6, IL-10, TNF, IL-1*β* and IL-12p40 in HCC cell-free supernatants were measured by using OptEIA Human ELISA Set (BD Biosciences) according to the manufacturer’s instructions.

### Statistical analysis

Besides studies using primary HCC tissues, all data are presented as means±S.D. of three independent experiments, with three replicates per sample/condition tested. Differences between averages were analyzed by two-tailed Student’s *t-*test. *P-*value of <0.05 was considered significant (*P<0.05, **P<0.01). Equal loading was confirmed by *β-*actin immunoblotting. For western blot analysis, the values below the figures represent relative density of the bands that were normalized to the corresponding *β-*actin signal. Target signals from immunoblottings were quantified by Scion Image software (Frederick, MD, USA). The acquired FACS data were analyzed with Summit software (Version 4.3.02, West Fargo, ND, USA).

## Figures and Tables

**Table 1:** Clinicopathological parameters in hepatocellular carcinoma (HCC)

**Patient**	**Age**	**Gender**	**Etiology**	**Cirrhosis**	**Tumor size**	**Microscopic VI**	**Macroscopic VI**	**Stage**
1	70	M	U	N	≥5 cm	Y	N	2
2	30	M	HBV	N	≥5 cm	Y	N	2
3	62	M	HBV	Y	<5 cm	Y	Y	2
4	68	M	HBV	N	≥5 cm	N	N	3A
5	52	M	HBV	Y	≥5 cm	N	N	3B
6	56	M	U	N	≥10 cm	Y	Y	3B
7	53	M	HBV	N	<5 cm	Y	N	2
8	64	M	HBV	N	≥5 cm	Y	N	2
9	66	M	HBV	Y	≥5 cm	Y	N	3A
10	70	F	HBV	N	<5 cm	Y	N	3B
11	66	M	HBV	N	≥10 cm	Y	N	3C

Abbreviations: M, male; F, female; HBV, hepatitis B virus; U, unknown etiology; VI, vascular invasion.

To better understand the critical regulator(s) in liver cancer, we retrospectively studied 11 HCC samples and their paracancerous normal liver tissues. Clinicopathological information provided from medical records includes age, gender, etiology, cirrhosis status, tumor size, types of vascular invasion and staging of the HCC patients by clinical TNM (tumor–node–metastasis).

## Abbreviations

HBsAg: hepatitis B surface antigen
HCC: hepatocellular carcinoma
SAG: sensitive to apoptosis gene
SARM: sterile *α* and HEAT/armadillo-motif-containing protein
UPS: ubiquitin–proteasome system.
